# Differentiation of anterior chamber pigment and inflammatory cells using swept-source optical coherence tomography: a cross-sectional study

**DOI:** 10.1038/s41433-025-03697-2

**Published:** 2025-03-12

**Authors:** Alice Bellchambers, Rongling Shu, Colin J. Chu, Harry Petrushkin, Ameenat Lola Solebo

**Affiliations:** 1 Population, Policy and Practice Programme, UCL GOS Institute of Child Health, London, UK; 2 East Sussex Healthcare NHS Trust, Eastbourne, UK; 3 Moorfields Eye Hospital NHS Trust, London, UK; 4 National Institute for Health Research Moorfields Biomedical Research Centre, London, UK; 5 UCL Institute of Ophthalmology, London, UK; 6 Great Ormond Street Hospital for Children NHS Trust, London, UK

**Keywords:** Uveal diseases, Biomarkers, Outcomes research

## Abstract

**Background/Objectives:**

We aimed to investigate the potential of anterior segment OCT (AS-OCT) in differentiating anterior chamber (AC) pigment and inflammatory cells.

**Subject/Methods:**

Cross-sectional study of adults with uveitis. The exclusion criterion was corneal opacity sufficient to obscure slit lamp examination of the anterior chamber. Reference testing comprised slit lamp-based detection of pigment and Standardization of Uveitis Nomenclature (SUN) grading of intraocular inflammation. Index testing comprised CASIA2 swept-source AS-OCT acquisition, with semi-automated analysis to detect and measure hyper-reflective particles within the AC. Correlations between AS-OCT-derived counts of different-sized particles and clinical grades were explored using multilevel multivariable regression analyses.

**Results:**

62 eyes (31 patients) were included. There was a positive correlation between AS-OCT particle counts of >4 pixels (equivalent to >24microns), and SUN grading (adjusted coefficient, adjCoef 24.3, 95% confidence interval 6.3 to 42.3, *p* = 0.03), strengthened in eyes clinically absent of pigment (adjCoef 20.6, 14.8 to 26.4, *p* < 0.001). A positive correlation was also noted between particle counts ≤2 pixels and the presence of AC pigment clinically.

**Conclusions:**

Swept-source (SS) AS-OCT holds potential utility in differentiating between pigment and cells within the AC, leading to improved management of individuals with or at risk of intraocular inflammation. SS AS-OCT-derived biomarkers may also provide information on uveitis aetiology, supporting the diagnosis of underlying conditions. Further work on a larger cohort, replication by other investigators and clinical teams, and clinical correlation with anterior chamber sampling will enable future clinical validation.

## Introduction

Uveitis encompasses a multitude of ocular inflammatory conditions with varying aetiologies, affecting a diverse population. The resultant visual and general health morbidity, impact on quality of life, socioeconomic burden, and demand on healthcare resources, are significant [1–4]

The current ‘gold standard’ clinical assessment for anterior uveitis, the most common form of the disease, is slit lamp examination with grading of intraocular inflammation using the Standardisation of Uveitis Nomenclature (SUN) criteria [5]. However, the clinical utility of this assessment is limited by subjectivity, intra- and inter-observer variability [6], and significant healthcare resource consumption, with the assessment relying on the skills of trained/qualified healthcare professionals, often within a hospital setting.

Anterior segment optical coherence tomography (AS-OCT) is an emerging diagnostic and monitoring tool for anterior uveitis, with the ability to provide objective and sensitive assessments of anterior chamber (AC) inflammation via the detection of hyper-reflective bodies on cross-sectional images [7, 8]. It is widely available and has the potential for widespread, community-based monitoring, with a subsequent reduction in the aforementioned uveitis-associated burden, on both individuals and healthcare providers.

To optimise the utility of AS-OCT for disease diagnosis and monitoring, it is important to identify the ‘true’ origins of the anterior chamber hyper-reflective bodies visible in AS-OCT images. Whilst there is evidence from animal, pre-clinical and clinical studies to support the widely held conclusion that these particles are inflammatory white blood cells [9–11], it is also widely recognised that there are other bodies within the AC, including pigment fragments [12, 13], which may present in a similar way on AS-OCT images [14, 15].

We sought to investigate the potential of AS-OCT images in differentiating between pigment and cells within the AC. We hypothesised that there would be an association between the size of the hyper-reflective bodies identified on AS-OCT and the differential presence of pigment and inflammatory cells within the AC on clinical examination. We sought to describe, specifically, the correlation between AS-OCT-derived cell count and clinical inflammatory grading in the presence and absence of clinically determined AC pigment. We also sought to quantify ‘small’ particles within AS-OCT images of eyes judged to have a high load of AC pigment on clinical examination.

## Subjects and Methods

This cross-sectional prospective study recruited adults managed within a single tertiary care centre. Eligible adults were those with a diagnosis of uveitis, or the presence of intraocular inflammation, with or without an additional diagnosis in which pigment manifests within the anterior chamber, specifically recent (within the last 3 months) intraocular surgery or non-penetrating ocular trauma. Individuals with corneal opacity judged (by their managing ophthalmologist) sufficient to obscure slit lamp assessment of the anterior chamber were excluded from the study.

### Reference testing

Adults underwent dilated slit lamp examination undertaken by at least two uveitis specialists. Slit lamp graders were blinded to index test findings. Inflammation of the AC was assessed using the standardisation of uveitis nomenclature (SUN) grading system, where 0 cells within a 1 mm x 1 mm beam are equivalent to 0 SUN, 1–5 cells =0.5 + SUN, 6-15 cells =1 + SUN, 16–25 cells = 2 + SUN, 26–50 cells =3 + SUN, and >50 cells =4 + SUN [16]. The presence of pigment in the anterior chamber was graded clinically using a three-step scale developed for this study: no pigment visible within a 1 mm x 1 mm slit lamp beam = ∠0, occasional pigment = 1+, more than occasional pigment = 2+.

### Index testing

The index test comprised image acquisition from dilated eyes using the CASIA2 (Tomey Corporation, Nagoya, Japan) swept-source optical coherence tomography platform. A standardised acquisition protocol (supplement) was used for all participants by the 2 eye health professionals (RS, CC) responsible for image acquisition. A set of 3 cross-sectional images (providing a volume of 12 mm length horizontally, 2 mm vertically, with the central image bisecting the pupil centre, and capture of the whole AC depth) was acquired from each eye in a darkened room (background illumination not measured). Images were extracted from the platform and converted into PNG format for semi-automated analysis. Data from all eyes of recruited participants were used, irrespective of the uveitis status of individual eyes (i.e. data from both eyes were used in participants with unilateral uveitis). Analysis methods have previously been described [14] and (in brief) comprised manual assessment of image quality [17] and manual drawing of a region of interest, specifically the anterior chamber, with automated image thresholding and detection of hyper-reflective signals within the chamber (using the ImageJ isothreshold and particle count functions) [18]. The identified hyper-reflective particles were further quantified using the maximal, minimum, and average brightness and size (in pixels) of each particle, with 1 pixel found to equate to approximately 6 microns (supplement).

### Analysis

Descriptive statistics were used for index test-derived metrics (counts of hyper-reflective particles and size and brightness of particles) and reference test-derived metrics (anterior chamber cell, flare and pigment grades). The repeatability of imaging-based metrics was explored using regression and Bland-Altman analyses. Different thresholds for particle size were used to interrogate possible associations of particle size with intraocular body ‘type’ (i.e. smaller pigment clumps versus larger inflammatory cells), specifically 1, 2, 3, 4 and 5 pixels in size. Counts of particles of sizes smaller than and equal to, or larger than, those thresholds were generated. Correlations between those derived particle counts and clinical grades were reported using multilevel kernel-based non-parametric regression analyses (i.e. with adjustment for any within-eye correlation where data were used from both eyes of individuals) with 95% confidence intervals based on bootstrap estimates. Receiver-operated characteristic curves were plotted to explore the impact of different thresholds on sensitivity and specificity for the outcome of the ‘gold standard’ reference test (routine examination with slit lamp) with robust regression used to adjust for the hierarchical nature of ‘both eyes’ data. Statistical analyses were undertaken in Stata (Stata, version. 17.0; StataCorp) and RStudio (RStudio, PBC, Boston, V2022.7.2.0).

This study received the necessary research approvals from the UK’s Health Research Authority (South Central Oxford B Research Ethics Committee, reference 19/SC/0283, amendment SA03). Written informed consent was obtained from all subjects.

## Results

### Description of the study cohort

Images were acquired from 62 eyes of 31 patients (Table 1). Of the 31, 12 were female (39%) and median age was 43.5 years (range 25–76 years). Almost half of the patients were of White British ethnicity (15, 48%), with 11 (35%) from Asian British backgrounds (including 4 patients from India, and 4 from Pakistani/Bangladeshi backgrounds), 4 (13%) from Black British backgrounds, and the remaining patients having mixed heritage or other backgrounds. Diagnoses included HLA-B*27/spondyloarthropathy-related uveitis (8, 26%) and idiopathic isolated anterior uveitis (7, 22%). Of the 31 participants, 12 had unilateral uveitis.

**Table 1:** Participant details.

Diagnosis	Right eye		Left eye	
	Pigment	AC activity	Pigment	AC activity
***Anterior uveitides***				
HLA-B*27 AAU +/− Ankylosing spondylitis, *n* = 8	0–1+	0–4+	0–1+	0–3+
Idiopathic isolated AAU/CAU, *n* = 8	0–1+	0–1+	0–1+	0–2+
Post phaco iatrogenic inflammation, previous history of uveitis, *n* < *5*	1+ – 2+	0.5 + –1+	0–0	0–0
Post phaco no previous history of uveitis, *n* < *5*	0–2+	0–0.5+	0–1+	0–0
Viral A/CAU, *n* < *5*	0–1+	0–1+	0–1+	0–1+
Other anterior uveitides^a^, *n* = *5–8*	0–2+	0–3+	0–2+	0–2+
***Other uveitides***				
Panuveitis, *n* < *5*	1+	1+	0	0.5+
Acute retinal necrosis, *n* < *5*	2+	0	0	0

Ranges have been provided for small numbers to avoid disclosure.

*AAU* acute anterior uveitis; *CAU* chronic anterior uveitis; *HLA* human leucocyte antigen; *Phaco* Phacoemulsification cataract surgery.

^a^Other anterior uveitides include: Idiopathic sclerouveitis; associated inflammatory bowel disease; associated sarcoidosis; post anti-vascular endothelial growth factor injection; post blunt trauma; post retained corneal metal foreign body.

### Reference test (clinical assessment) findings

Of the 62 eyes, 32 (52%) were deemed 0 SUN, 0.5 + SUN was noted in 11 eyes, 1 + SUN in 10, 2 + SUN = 6, and 3 eyes were 3 + SUN/4 + SUN. The pigment was absent in 37 eyes (60%), 22 eyes (35%) had occasional pigment, and the remaining 3 eyes (5%) had more than occasional pigment in the anterior chamber. Of the 25 eyes with pigment, 9 (36%) were deemed free of AC cells, and of the 30 eyes with >0 SUN, 14 eyes were free of pigment (47%), with no evidence of a statistically significant correlation between AC cell and pigment load (correlation *r*^2^ = 0.09; multilevel ordinal logistic regression of AC cell grade with pigment level with adjustment for within-patient clustering adjusted odds ratio, aOR = 1.39, *p* = 0.34, 95% confidence interval, CI = 0.70–2.76). Exploration of associations between demographic factors and pigment or cell status revealed no association with age or gender. The pigment was less likely to be present in patients with White British ethnic backgrounds (pigment-free eyes in 57% of White British patients versus 27% of Asian, 16% of Black, and 0% of patients from other Ethnic backgrounds), but this difference was not statistically significant (Pearson chi^2^(6) = 11.3, *p* = 0.08).

### Index test (AS-OCT) findings

**Fig. 1:**
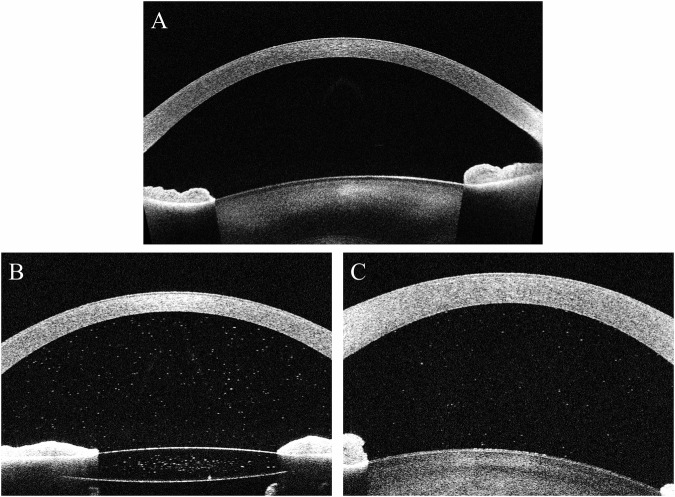
Example of AS-OCT acquired from eyes with and without clinical activity and with and without anterior chamber pigment. **A** Clinically inactive eye free from pigment; **B** Clinically inactive (SUN0.5+) pseudophakic eye with anterior chamber pigment; **C**. Clinically active SUN2+ eye free of pigment.

Analysis of cross-sectional images acquired from participants resulted in particle counts ranging from 0–4389 particles per image, with a median of 2 particles per image (Fig. 1). Particle brightness scores (where 100=maximal brightness and 0 = black) ranged from 59.5 to 93.7 (median 75.5). Repeatability indices for scans taken from individual eyes (derived through comparison of horizontal scans separated by 1 mm), revealed tight within-eye correlation for particle counts, particle density (counts corrected for across different horizontal locations using areas of region of interest), and particle sizes (correlation coefficient adjusted for within-patient correlation 0.97, *p* < 0.001, 95% confidence interval 0.96–0.99 for count; 0.99, *p* < 0.001, 95% confidence interval 0.98- 0.99 for density; 0.70, *p* < 0.001, 95% confidence interval 0.46–0.92 for size; 0.79, *p* < 0.001, 95% confidence interval 0.57–1.00 for brightness). Bland Altman (BA) bias-adjusted plots confirmed reasonable repeatability cell count, particle size and particle brightness (BA plots available as Supplemental Fig.  S1A–S1C).

### Correlation of clinical assessment with image metrics

**Fig. 2:**
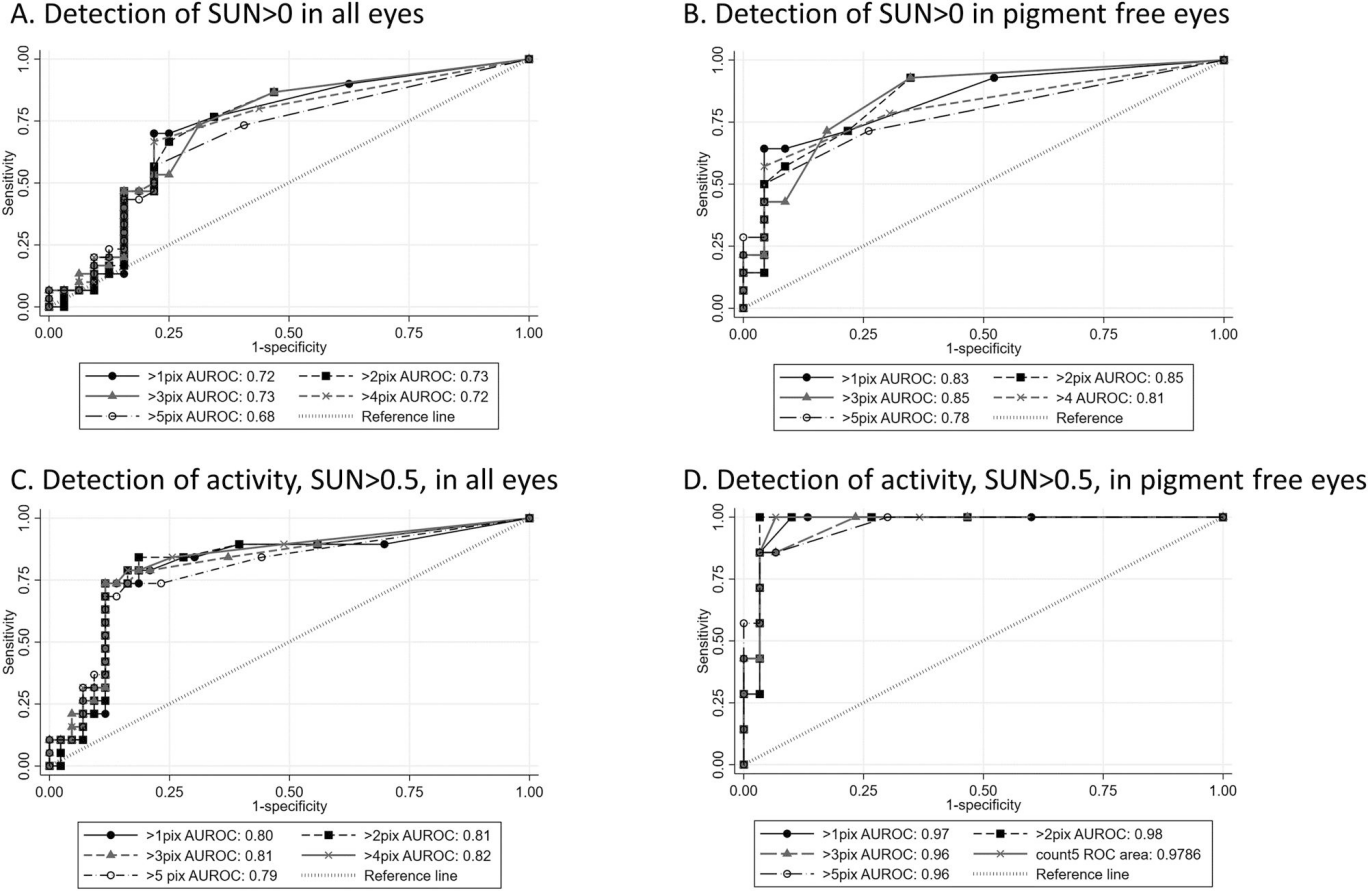
ROC curves for diagnostic performance of particle count. Receiver operated characteristic curves for diagnostic performance of particle count with different size thresholds in all eyes and in eyes clinically free of anterior chamber pigment.

Correlation indices identified a positive relationship between particle counts and anterior chamber cell grade, with strengthened associations in eyes clinically absent of pigment (Table 2). Median particle size for eyes with clinical activity (SUN > 1+), which were free of pigment, was 7.4 (range 5.8–8.9), whilst the median size for inactive size, which were deemed to have pigment within the AC, was 4.2 (range 2–12). Across the receiver-operated characteristic curves, there was a higher area under the curve (AUROC) with the use of a threshold of >2 pixels to identify clinically active levels of disease (Fig. 2), again with evidence of improved performance of AS-OCT detection of clinically graded aqueous cellular burden in eyes free of pigment. There was no association between ethnicity or gender and AS-OCT-derived particle counts.

**Table 2:** Association with increasing SUN score for anterior chamber cells.

	Adjusted correlation indices, 95% confidence interval, *p*	
	All eyes	Eyes free of pigment
Particle count	−144.4 −505.2 to 216.4 *p* = 0.43	28.1 20.3 to 35.8 *p* < 0.001
Particle count, threshold 2 pixels	−69.1 −252.8 to 114. 6 *p* = 0.46	26.9 19.6 to 34.3 *p* < 0.001
Particle count, threshold 3 pixels	26.1 1.2 to 50.9 *p* = 0.12	23.9 16.8 to 31.1 *p* < 0.001
Particle count, threshold 4 pixels	24.3 6.3 to 42.3 *p* = 0.03	20.6 14.8 to 26.4 *p* < 0.001
Particle count, threshold 5 pixels	21.2 7.3 to 35.1 *p* = 0.003	17.9 12.7 to 23.0 *p* < 0.001

**Fig. 3:**
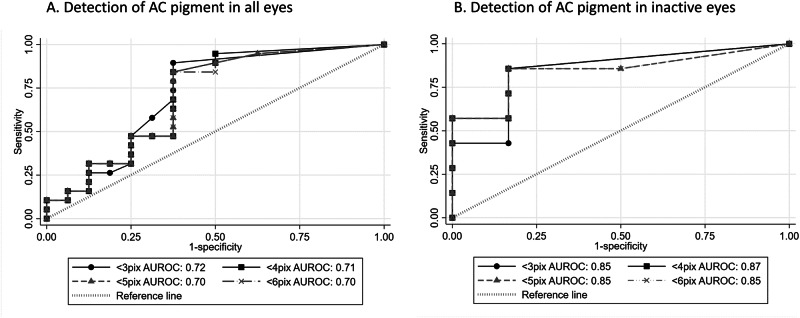
ROC curves for diagnostic performance of small particle count. Receiver operated characteristic curves for diagnostic performance of small particle count using different particle size thresholds in (**A**) all eyes and (**B**) eyes clinically free of inflammatory cells.

The correlation of counts of small particles and clinically assessed presence of AC pigment did not reach statistical significance, (adjusted correlation between presence of pigment and counts for cells ≤2 pixels in inactive eyes = 17.4, 95% CI −4.2 to 39.5, *p* = 0.1), but there was evidence of some diagnostic utility with the greatest area under the curve achieved using a small particle threshold of less than 3 pixels in size (Fig. 3).

### Correlations between imaging metrics and diagnosis

There were statistically significant associations between diagnosis type and average particle size on anterior segment OCT. In clinically active eyes, when compared to a ‘baseline’ diagnosis of recurrent idiopathic isolated recurrent acute anterior uveitis, particle size was smaller in eyes with viral uveitis (coefficient for size in pixels adjusted for clinical detection of pigment, and within individual clustering = −0.15, *p* < 0.05, 95% CI −0.01 to −0.29, Supplemental Fig. S2).

## Discussion

From this cross-sectional study of a representative sample of adults with a diagnosis of uveitis, we improved the correlation of AS-OCT-derived particle count with clinical grading of inflammation in the absence of AC pigment. Conversely, we report that AS-OCT-derived counts of smaller particles (<3 pixels size on CASIA2 swept source imaging, equating to particles approximately <18 microns in diameter) appeared to hold some sensitivity and specificity for the detection of clinically significant AC pigment, particularly in eyes free of clinically active uveitis. These findings suggest that AS-OCT imaging metrics hold utility in the differentiation of pigment and cells within the anterior chamber.

Previous investigators have described the presence of AS-OCT-derived anterior chamber particles following laser iridotomy [19] or selective laser trabeculoplasty [20] but have not sought to identify the biological origin (inflammatory cell, red blood cell, pigment or other) of these identified particles. Multiple studies have found that AS-OCT detects AC cells as hyper-reflective spots on cross-sectional images, which correlate well with clinical anterior chamber inflammatory cell grading [10, 21, 22]. Our report is consistent with those of earlier investigators, but our findings also report the impact of AC pigment and particle size thresholding on diagnostic performance.

The utility of AS-OCT as a uveitis diagnostic and monitoring tool relies on its sensitivity and specificity for detecting inflammatory cells within the AC. Sensitivity, particularly with swept-source modalities, has been consistently reported as high [7, 8]. In an attempt to improve specificity (i.e. lowering the ‘false positive rate’ of AS-OCT versus clinical assessment as a gold standard comparator), previous studies have applied thresholds based on particle size for quantitative analysis of cell count in acquired AS-OCT images [10, 23]. This approach was postulated to enable the differentiation of inflammatory cells from background ‘noise’ or ‘clinically irrelevant’ anterior chamber findings. Our study supports this thresholding approach but also suggests that the smaller particles detectable on AS-OCT may represent pigment within the AC [10, 14, 21]. The findings of improved performance of cell detection in the absence of pigment, and vice versa, also suggest that the work currently underway to develop and standardise image analysis protocols will need to consider the applicability of AS-OCT imaging approaches for anterior chamber inflammation across different clinical conditions. Algorithms developed for AC cell quantification in anterior uveitis may not be transferable to eyes that also have anterior chamber pigment. This will include individuals with inflammatory changes following intraocular procedures.

The potential utility of AS-OCT imaging of anterior chamber contents beyond the simple quantification of inflammatory cells has also been suggested by others. Keratic precipitates, thought to hold some diagnostic utility for uveitis type [13], have been characterised by AS-OCT [13, 26]. The size of the anterior chamber particles noted on AS-OCT may also hold diagnostic utility for disease characterisation. Early reports from in-vitro work on the correlation of AS-OCT particle size and brightness with leucocyte type suggest that AS-OCT-derived biomarkers for inflammatory cell population characterisation are feasible [11]. Anterior chamber leucocyte population characteristics have been shown to indicate disease type, with, for example, patients with HLA-B27 uveitis having a predominantly polymorphonuclear anterior chamber cell population, whilst those with sarcoidosis have a predominantly mononuclear pattern [11, 13, 24, 25]. Larger AC particles have been identified in AS-OCT images acquired from patients with JIA, when compared to eyes with ocular sarcoid [26], or to idiopathic isolated chronic anterior uveitis. [14] Our findings also suggest that AS-OCT may be able to differentiate uveitis aetiology, with smaller particle sizes noted in viral uveitides versus non-infectious diseases. However, in the absence of other work from external populations, our postulated correlations between underlying systemic diagnosis and AS-OCT-derived measurements of anterior chamber particle size remain unsupported. Future research should involve taking advantage of episodes of aqueous sampling to examine the correlates of AS-OCT image metrics and aqueous cell population. Investigators should also consider other clinical predictors of cell features, with, for example, the evaluation of correlations between disease chronicity and cell size. The advent of the high-speed swept source OCT instruments (with a typically higher axial resolution when compared to spectral domain platforms) has enabled this more precise capture of cell characteristics. Future improvements in scanning modalities should provide further advances in a non-invasive examination of intraocular immune cell type.

Rose-Nussbaumer et al hypothesised that AS-OCT-derived particle metrics, including axial width and reflectance measurements, are derived from different parts of the cell, dependent on cell type; leucocyte cytoplasm has a similar refractive index to the surrounding aqueous humour and thus its metrics are derived from the cell nuclei rather than the whole cell, compared to anuclear red blood cells, where the haemoglobin within the cytoplasm renders the refractive index sufficiently different from surrounding aqueous, and thus the measurements are derived from the entire cell [11]. It has been suggested that AC pigment is composed of melanin, with a high refractive index [27], and composed of nanoparticles measuring 30-50 nm only [27]; far smaller than the 6 microns required for it to be detected as a 1 pixel size particle on AS-OCT. This may suggest that some AC pigment is not ‘free’ melanin, but rather, melanin within a larger body, although agglomeration of pigment particles within the AC is feasible. A larger dataset of AS-OCT images of AC pigment is needed to test this hypothesis.

Further research is required to interrogate whether there is a difference in particle brightness between pigment and inflammatory cells on AS-OCT, which may aid the development of a brightness threshold metric for the detection of AC cells. The absence of a statistically significant difference between mean particle brightness and clinical grading of cell or pigment presence in our study may have been due to the relatively small sample size presented here, and by the omission of patients free of uveitis who had only AC pigment clinically.

A key limitation of this study is the subjectivity of the reference test. Despite being the current ‘gold standard’ assessment for anterior uveitis, slit lamp examination and SUN grading is a subjective measurement, limiting their validity and utility as a reference test. The use of 2 independent graders aimed to reduce this limitation. However, replication of our work by other investigators and/or the use of an objective reference test will be important for the validation of our findings. Laser flare photometry (LFP), the only current, objective, validated metric for assessing AC flare, a measure of AC inflammation, was not used in this study. Its use in future work may build upon the findings presented here. However, LFP is not validated for the assessment of pigment. The study is also limited by the absence of an agreed standard for the grading of AC pigment clinically. Clinical evaluations such as examination of the corneal endothelium or gonioscopy can be used to identify pigment deposits within the anterior chamber but will not provide a quantification of pigment distributed within the aqueous.

In summary, our study has found that AS-OCT-derived metrics hold utility in differentiating between cells and pigment within the anterior chamber and may provide additional information regarding uveitis aetiology. With the current shortage of eye health professionals worldwide, there is a need for innovative approaches, including the implementation of high-quality community-based care. This relies on objective, sensitive tools, such as AS-OCT. The case for clinical utility will be built upon findings such as those presented here. Further work will involve higher-powered studies and replication of our findings by other investigators.

## Summary

### What was known

- Anterior segment optical coherence tomography (AS-OCT) is able to provide objective and sensitive assessments of anterior chamber (AC) inflammation, via the detection of hyper-reflective bodies, which are presumed to be leucocytes
- Similar hyper-reflective dots have been noted on AS-OCT imaging of individuals with anterior chamber pigment.

### What this study adds

- There is a correlation between AS-OCT particle size and clinical findings, with larger particles correlating with AC inflammation and smaller particles correlating with AC pigment
- These findings support the future use of AS-OCT in differentiating these clinical states.

## Supplementary information

Supplemental figures 1A-C

Supplemental figure 2

## Data Availability

The datasets analysed during the current study are available from the corresponding author on reasonable request, and subject to the requester organising the necessary governance approvals and institutional data-sharing agreements.
