# Supplementary material for: Differentiation of anterior chamber pigment and inflammatory cells using swept-source optical coherence tomography: a cross-sectional study
Source: Eye (Lond). 2025 Mar 12;39(9):1744–50. doi: 10.1038/s41433-025-03697-2 (PMC12130485; doi:10.1038/s41433-025-03697-2)

Supplement Figure S1A-C. Bland Altman (BA) plots showing strong repeatability of cell count, particle size and brightness across cross-sectional images of same eye

Dashed line = central agreement line; dotted line = 95% limit of agreement, unbroken grey line = 99% limit of agreement

S1A: BA plot of particle count repeatability, units=particles per cross sectional image

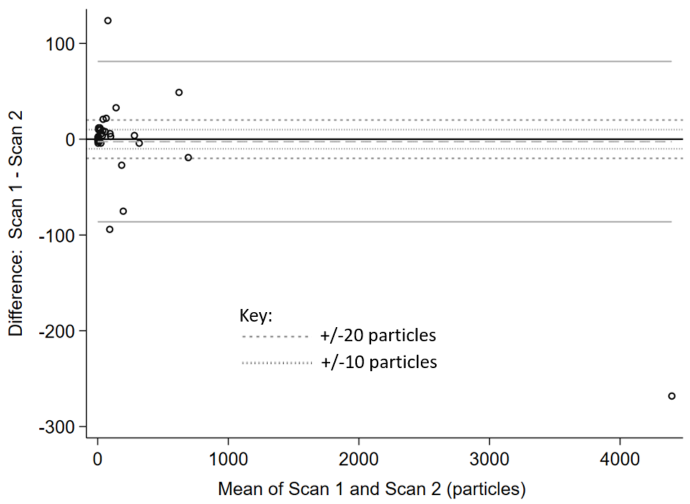

S1B: BA plot of particle size, units=size in pixels

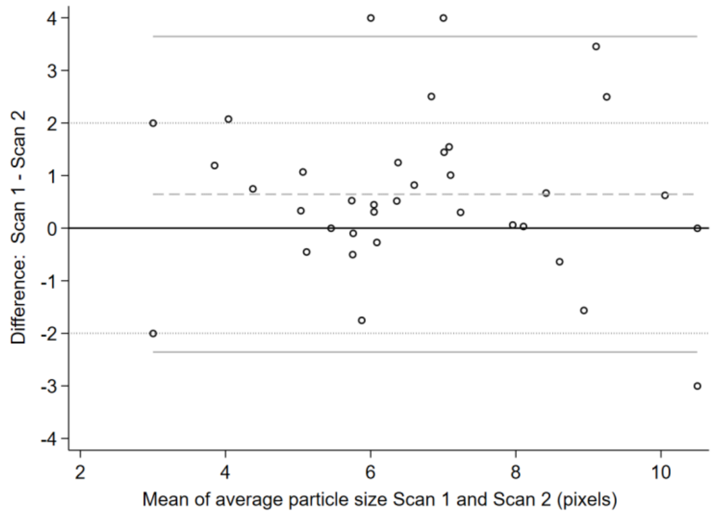

S1C: BA plot of particle brightness, units=brightness on luminance scale from 0 (darkest) to 100 (brightest)

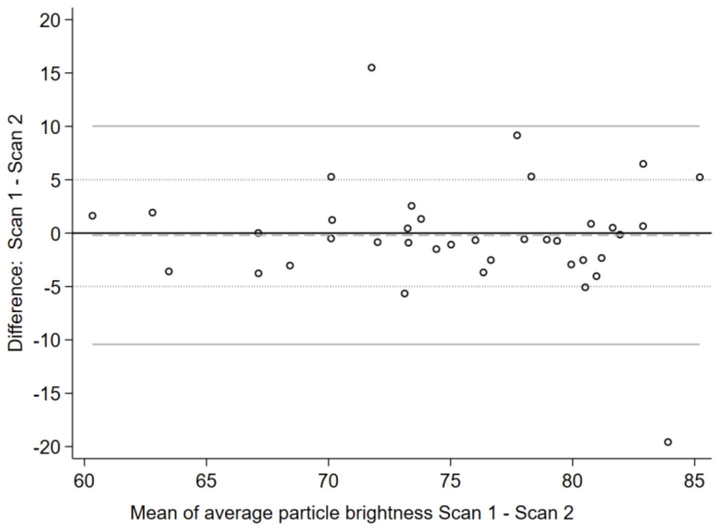

Supplement: Supplementary file 1 — Supplemental figures 1A-C [file 41433_2025_3697_MOESM1_ESM.pdf]
