# Supplementary material for: Differentiation of anterior chamber pigment and inflammatory cells using swept-source optical coherence tomography: a cross-sectional study
Source: Eye (Lond). 2025 Mar 12;39(9):1744–50. doi: 10.1038/s41433-025-03697-2 (PMC12130485; doi:10.1038/s41433-025-03697-2)
Supplement: Supplementary file 2 — Supplemental figure 2 [file 41433_2025_3697_MOESM2_ESM.pdf]

Supplemental figure S2: Average particle sizes (unadjusted) within cross-sectional scans by patient diagnosis

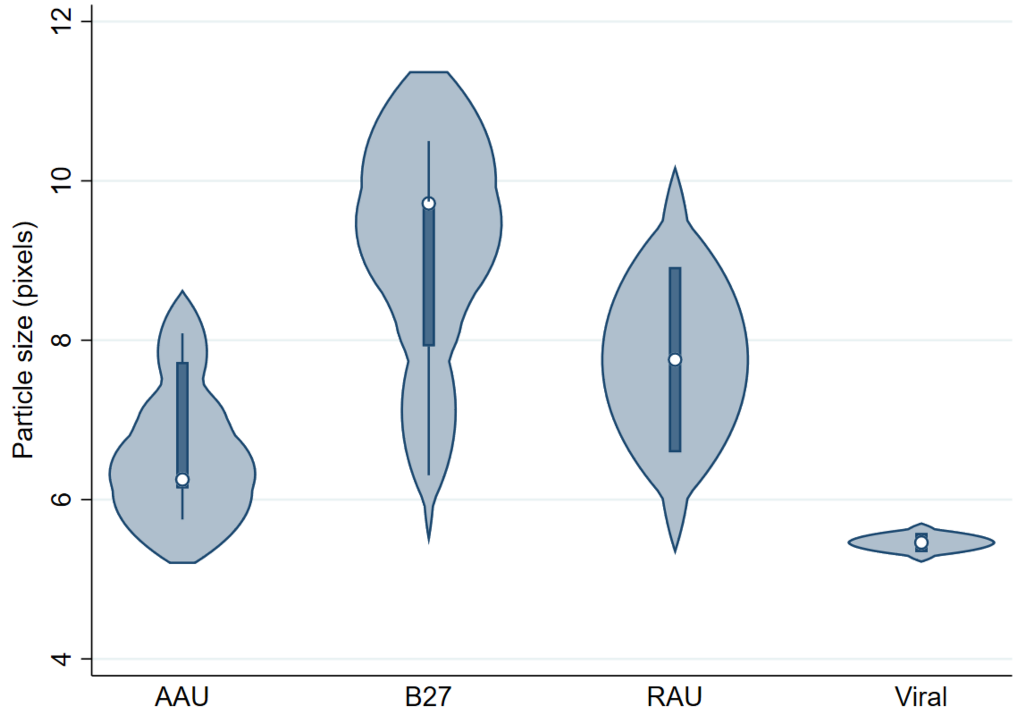

AAU=Acute anterior uveitis
B27= Human leukocyte antigen B27 positive uveitis
RAU='Reactive' (iatrogenic post surgical) anterior uveitis
